# Recurrent *Campylobacter jejuni* bacteraemia with independent acquisition of carbapenem resistance in three ST9611 isolates: a case series in immunocompromised hosts

**DOI:** 10.1186/s13099-026-00833-5

**Published:** 2026-04-09

**Authors:** Jinghao Nicholas Ngiam, Matthew Chung Yi Koh, Grace Tung, Fengjie Tang, Jeanette Teo, Ka Lip Chew

**Affiliations:** 1 Division of Infectious Diseases, Department of Medicine, National University Health System, 1E Kent Ridge Rd, NUHS Tower Block, Level 10, 119228 Singapore, Singapore; 2 Department of Laboratory Medicine, National University Hospital, 5 Lower Kent Ridge Road, Singapore, 119074 Singapore

**Keywords:** Carbapenem resistance, Campylobacter, Bacteraemia, Bloodstream infection, Immunocompromised

## Abstract

**Objectives:**

*Campylobacter jejuni* is a leading cause of bacterial gastroenteritis but rarely causes bacteraemia, except in immunocompromised hosts. Carbapenem resistance in *C. jejuni* remains uncommon, and the clinical evolution of resistance during infection has not been well described.

**Methods:**

We report a case series of three immunocompromised adults with recurrent *C. jejuni* bacteraemia at a tertiary hospital in Singapore between 2024 and 2025.

**Results:**

All three patients had underlying haematologic or autoimmune disorders associated with hypogammaglobulinaemia. Each experienced multiple episodes of *C. jejuni* bacteraemia despite prolonged carbapenem therapy, with isolates showing stepwise increases in meropenem minimum inhibitory concentrations (MICs) from ≤ 0.05 mg/L to as high as 16 mg/L. No abscesses or drainable foci were identified radiologically. Two patients received regular intravenous immunoglobulin (IVIG) replacement after low serum IgG levels were detected. In all cases, recurrences ceased after introduction of eravacycline therapy, with or without concurrent oral gentamicin, in combination with IVIG support. One patient later died of progression of underlying lymphoma.

**Conclusions:**

This series demonstrates the potential for acquired carbapenem resistance to develop in *C. jejuni* during recurrent bacteraemia in immunocompromised hosts. Eravacycline, alongside immunoglobulin replacement, may represent a viable therapeutic option in such refractory infections.

**Supplementary Information:**

The online version contains supplementary material available at 10.1186/s13099-026-00833-5.

## Introduction

*Campylobacter jejuni* is an important cause of bacterial gastroenteritis worldwide but an uncommon cause of bacteraemia, typically occurring in immunocompromised hosts such as those with haematologic malignancies or hypogammaglobulinaemia [1, 2]. Increasing resistance to macrolides and fluoroquinolones in *C. jejuni* has been reported. This may be a result of selective pressure from antimicrobial use in both humans and animals. Carbapenems are generally regarded as reliably active against *C. jejuni* and are typically used in refractory or invasive infections such as bacteraemia. Nevertheless, acquired carbapenem resistance remains exceptionally rare, with only a few isolated cases documented in the literature [3, 4]. 

Carbapenem resistance in *C. jejuni* has been linked to multiple mechanisms, including upregulation or mutation of the chromosomal bla_OXA−61_ gene, alterations in outer membrane porins, and efflux pump overexpression [5]. Here, we describe three immunocompromised patients with recurrent *C. jejuni* bacteraemia who developed carbapenem resistance during their clinical course. All exhibited deficiency in humoral immunity and suffered multiple recurrences despite prolonged carbapenem therapy, with eventual resolution following the introduction of alternative agents such as eravacycline. This series highlights the potential for *C. jejuni* to cause recurrent bacteraemia and acquire carbapenem resistance in vivo (Table 1). Additional details on laboratory phenotypic and genotypic testing are available in the supplementary appendix.

**Table 1:** Clinical characteristics of three immunocompromised patients with acquired carbapenem resistance in *Campylobacter jejuni* bacteraemia

Patient age/sex	Past medical history	Infectious episode number	Culture date	Isolate WGS number	MERO	TET	ERV	Potential meropenem resistance mutations	Antimicrobial treatment		Other notes
									Drugs	Duration (days)	
Patient 1 74/Male	Relapsed aggressive large B-cell lymphoma, autologous stem cell transplant on 10/5/2023; received epcoritamab thereafter	1	16/2/24	CAM11	0.5	N/A		-	Meropenem (IV) Ertapenem(IV)	5 9	IgG 4.2, IVIG approximately 3-weekly as needed
		2	11/12/24	CAM12	0.06			-	Meropenem (IV)	14	
		3	02/01/25		0.25				Ertapenem (IV)	14	
		4	06/02/25	CAM01	0.016			-	Meropenem Ertapenem (IV)	14 14	
		5	27/03/25	CAM02	0.125			-	Meropenem (IV) Eravacycline (IV)/ Gentamicin (PO)	10 28/42	
			31/03/25	CAM03	1			PorA D311N			
			02/04/25	CAM04	4	> 256	0.016	PorA D311 PBP3 G584E N			
Patient 2 39/Male	Systemic lupus erythematosus with lupus nephritis, on haemodialysis, on hydroxychloroquine 200 mg 3x/week and prednisolone 5 mg daily; with common variable immunodeficiency	1	04/04/25	CAM05	0.5	N/A		-	Meropenem (IV)	10	IgG < 1.1, continued on monthly IVIG
		2	22/05/25	CAM07	0.5	N/A		-	Meropenem (IV)	7	
		3	23/06/25	CAM09	16	> 256	0.016	PorA D308Y	Eravacycline (IV)	14	
Patient 3 62/Male	Waldenstrom’s macroglobulinaemia; EBV positive large B cell lymphoma treated with ritixumab, cyclophosphamide, vincristine and doxorubicin	1	26/05/25	CAM8	0.5	N/A		-	Meropenem (IV)	7	IgG 4.4, IVIG given Died from underlying disease 1 month later
		2	07/07/25	CAM10	8	0.5	0.008	OXA-193 D201G PorA Q312K PBP3 V500I	Eravacycline (IV)	14	

WGS number input for reference, KIV remove in final submission

MERO: Meropenem; TET: Tetracycline; ERV: Eravacycline: AMR: Antimicrobial resistance; PBP2: penicillin-binding-protein-2; N/A: Not available; IV: Intravenous; PO: Per oral: IVIG: Intravenous immunoglobulin

All isolates were resistant to ciprofloxacin and erythromycin

### Case 1

A 74-year-old man with relapsed aggressive large B-cell lymphoma underwent autologous stem-cell transplantation in May 2023 and subsequently received epcoritamab therapy. He presented in February 2024 with fever and diarrhoea, and *Campylobacter jejuni* was isolated from blood cultures (meropenem MIC 0.5 mg/L). He was treated with intravenous meropenem for five days followed by ertapenem for nine days, but experienced recurrent episodes of *C. jejuni* bacteraemia over the next year, each separated by brief asymptomatic intervals. The initial episodes of bacteraemia were short-lived (~ 2 days), but in the final episode, the patient remained bacteraemic for ~ 8 days. Repeat isolates showed progressively increasing meropenem MICs (0.06 to 4 mg/L). Serial imaging, including PET-CT and MRI of the rectum, revealed no drainable focus. Despite multiple prolonged courses of carbapenems, recurrences persisted until March 2025, when he received a combination of IV eravacycline for four weeks and oral gentamicin for six weeks, together with regular IV immunoglobulin (IVIG) replacement for secondary hypogammaglobulinaemia (IgG 4.2–5.4 g/L). No further bacteraemic episodes occurred after initiation of eravacycline and IVIG.

### Case 2

A 39-year-old man with systemic lupus erythematosus and lupus nephritis on haemodialysis, maintained on low-dose prednisolone and hydroxychloroquine, had concomitant common variable immunodeficiency. He developed *Campylobacter jejuni* bacteraemia in April 2025 (meropenem MIC 0.38 mg/L) and was treated with 10 days of IV meropenem. His serum IgG level was < 1.1 g/L, and monthly IVIG was initiated. Despite this, he experienced two further recurrences in May and June 2025 with increasing meropenem MICs up to 16 mg/L. A gastrointestinal source of infection was suspected, as PET-CT imaging and colonoscopy during the second episode demonstrated FDG uptake and patchy erythema in the descending and sigmoid colon, consistent with enterocolitis. Subsequent CT scans showed diffuse colonic wall thickening without abscess formation. The final episode was managed with a 14-day course of IV eravacycline while IVIG replacement was continued. No further recurrences occurred thereafter.

### Case 3

A 62-year-old man with Waldenström’s macroglobulinaemia and Epstein-Barr virus-positive large B-cell lymphoma, previously treated with rituximab, cyclophosphamide, vincristine, and doxorubicin, presented in May 2025 with fever and diarrhoea. Blood cultures yielded *Campylobacter jejuni* (meropenem MIC 0.5 mg/L), and he was treated with a 7-day course of IV meropenem. He re-presented six weeks later with recurrent bacteraemia, and the isolate demonstrated an increased meropenem MIC of 8 mg/L. The infection source was uncertain, though an infected peripherally inserted central catheter which had been retained since the prior bacteraemia episode was deemed as possible source. Subsequent PET-CT imaging revealed no drainable or metastatic focus. He received a 14-day course of IV eravacycline and was administered IV immunoglobulin for hypogammaglobulinaemia (IgG 4.4 g/L). No further *C. jejuni* recurrences occurred, though he succumbed to progression of his underlying malignancy one month later.

### Putative resistance mechanisms and structural predictions of carbapenem resistance in Campylobacter spp.

Carbapenem non-susceptibility in *Campylobacter spp.* can be attributed to: (i) promoter mutations upstream of chromosomal blaOXA that up-regulate β-lactamase expression; (ii) blaOXA copy-number increases (gene duplication) and (iii) structural changes in PorA (major outer-membrane porin) that reduce carbapenem influx. In a recurrent *C. jejuni* bacteraemia under prolonged ertapenem therapy, the resistant recurrent isolate carried a PorA mutation together with promoter alterations and duplication of blaOXA-61.^4^ In another report, during meropenem therapy for persistent *C. coli* bacteraemia, resistance arose in vivo via a PorA point mutation plus overexpression of blaOXA-489 driven by promoter changes and gene-dose increase, and MICs partially regressed when the extra copy was later lost [3]. 

In our study, all isolates from patients on prolonged carbapenem therapy with elevated meropenem MICs carried PorA mutations clustered at residues 308–312 (Table 2). These substitutions occur within loop L6, a critical region influencing pore size and β-lactam permeability, where even modest ΔΔG shifts can alter charge distribution and side-chain orientation. These are factors well-recognized as determinants of carbapenem influx limitation [6]. The universal presence of PorA mutations across all isolates, combined with secondary substitutions in PBP3 (V500I, G584E) and the OXA_193_ D201G variant in another isolate, patient 3 (Table 2), may account for the development of carbapenem resistance.

**Table 2:** Isolates with raised meropenem MICs analyzed with respect to their susceptible index isolates

Isolate	MEM MIC (mg/L)	Protein and amino acid change	DynaMut2 ΔΔG (kcal·mol⁻¹)^A^	Predicted stability change (DynaMut2)^A^	FoldX ΔΔG (kcal·mol⁻¹)^B^	Predicted stability change (FoldX)^B^
**Patient 1**						
CAM03	1	PorA D311N	−1.11	Destabilising	−1.030	Stabilising
CAM04	4	PBP3 G584E	0.03	Stabilising	0.571	Destabilising
		PorA D311N	−1.11	Destabilising	−1.030	Stabilising
**Patient 2**						
CAM09	16	PorA D308Y	−0.55	Destabilising	2.8	Destabilising
**Patient 3**						
CAM10	8	OXA-193 D201G	−0.10	Destabilising	0.33	Destabilising
		PorA Q312K	−0.09	Nominally destabilising	−0.94	Stabilising
		PBP3 V500I	−0.28	Destabilising	0.34	Destabilising

ΔΔG values represent the difference in Gibbs free energy between the mutant and wild-type proteins (ΔΔG = ΔG_mut − ΔG_wt)

^A^DynaMut2: ΔΔG > 0 = stabilizing, ΔΔG < 0 = destabilizing

^B^FoldX: ΔΔG > 0 = destabilizing, ΔΔG < 0 = stabilizing

## Discussion

*Campylobacter jejuni* bacteraemia is an uncommon clinical entity, typically confined to immunocompromised hosts [1, 2]. Recurrent or relapsing episodes are also exceedingly rare [7, 8]. In this present series, all three patients experienced multiple recurrences over several months despite appropriate therapy. Each had an underlying condition associated with hypogammaglobulinaemia, suggesting that impaired humoral immunity may have facilitated incomplete bacterial clearance and persistence within the gastrointestinal tract, leading to a greater propensity for recurrence [7–10]. Pairwise SNP distance analysis of sequential isolates within each patient demonstrated close genetic relatedness, with low SNP distances observed between longitudinal isolates (Patient 1: 5–26 SNPs; Patient 2: 3–6 SNPs ; Patient 3: 6 SNPs). A pre-determined threshold was not used to determine genetic relatedness but thresholds up to 40 and 43 SNPs have been used due to heterogeneity in *C. jejuni* populations [11, 12]. While re-infection cannot be ruled out, these findings indicate a high likelihood of relapsing episodes of infection with a persistent strain of *Campylobacter* over time. The greater diversity seen in Patient 1 likely reflects the greater time periods between the bacterial isolates. Additionally, all three patients had isolates that were uniformly resistant to macrolides and fluoroquinolones, which precluded them as therapeutic options. These agents are able to achieve high intracellular concentrations compared with beta-lactams, which may be necessary for successful eradication of an intracellular pathogen like *C. jejuni* [13, 14]. In all three patients, imaging did not demonstrate any deep or undrained foci to account for the recurrence of infections. Stool testing was not routinely performed for each case as clinical management was guided primarily by blood culture results in the setting of invasive infection.

All isolates in this series belonged to sequence type ST9611 (Supplementary Fig. 1), a lineage rarely described in association with human infection [15]. Data from the PubMLST *Campylobacter jejuni/coli* database, which includes over 150,000 isolates globally, suggest that ST9611 represents a relatively uncommon sequence type. However, the apparent rarity of ST9611 may reflect geographic variation or under-sampling. We did not find any epidemiologic linkage among the patients to suggest nosocomial or clonal transmission, and all infections had been community acquired. The independent identification of ST9611 across three unrelated hosts raises the question of whether this genotype possesses distinct biological or virulence factors that predispose to persistent colonisation and elevated risk for recurrent infections. Further genomic and phenotypic comparisons with other *C. jejuni* lineages evaluating virulence markers and resistance genes will be required to clarify whether ST9611 harbours these unique properties.

Finally, the progressive rise in meropenem MICs observed during serial episodes in all three patients strongly suggests *in viv*o acquisition of carbapenem resistance under therapeutic exposure. Whole-genome sequencing revealed carriage of bla_OXA−61_-like alleles, with additional mutations identified in mrdA (encoding penicillin-binding protein 2) and outer membrane porins such as porA, which would likely contribute to elevated carbapenem MICs [3, 4]. Although we propose putative resistance mechanisms, these findings are based on genomic and in silico analyses without functional validation. The precise contribution of individual mutations to carbapenem resistance remains uncertain. Such development of resistance on therapy has also been previously described in a patient who had hypogammaglobulinaemia (Good’s syndrome) [3]. Although whole-genome sequencing allowed putative identification of resistance determinants, functional validation of the observed mutations was not performed, and their precise contribution to carbapenem resistance requires further confirmation. Nonetheless, our findings raise caution about *C. jejuni* as an emerging pathogen in immunocompromised hosts, demonstrating the propensity for recurrent infections, as well as the development of carbapenem resistance on therapy. In our three cases, there was successful clearance of infection following introduction of eravacycline as an alternative agent, in combination with IVIG. With eravacycline therapy, high intracellular drug concentrations can be achieved which may have contributed to more effective clearance of *C. jejuni.* In one case, oral gentamicin decolonisation strategy was used concurrently [16]. Effectiveness of the specific antibiotic choices also cannot be distinguished from concurrent interventions such as IVIG replacement, as well as the differences in the baseline host immune status. Combination antibiotic therapy has been used to treat other resistant Gram-negative bacterial infections, but its efficacy has yet to be studied in the context of *C. jejuni*. The optimal treatment strategy remains poorly-defined.

## Conclusions

This case series illustrates the potential for *C jejuni* to recur, and to acquire carbapenem resistance in immunocompromised hosts, likely driven by antimicrobial pressure and impaired humoral immunity. Early recognition of this phenomenon may be important, as adjunctive strategies such as IVIG and gastrointestinal decolonisation may help to achieve microbiological cure of this infection.

## Supplementary Information

Below is the link to the electronic supplementary material.

Supplementary Material 1.

## Data Availability

No datasets were generated or analysed during the current study.
