# Supplementary material for: Recurrent Campylobacter jejuni bacteraemia with independent acquisition of carbapenem resistance in three ST9611 isolates: a case series in immunocompromised hosts
Source: Gut Pathog. 2026 Apr 9;18:43. doi: 10.1186/s13099-026-00833-5 (PMC13220455; doi:10.1186/s13099-026-00833-5)
Supplement: Supplementary file 1 — Supplementary Material 1. [file 13099_2026_833_MOESM1_ESM.docx]

**Appendix: Phenotypic susceptibility testing and whole-genome-sequencing methods**

Isolates and antimicrobial susceptibility testing

Longitudinal patient isolates with raised meropenem MICs were compared with their corresponding meropenem-susceptible index isolates from the same patient. These isolates were identified as Campylobacter jejuni using Matrix-Assisted Laser Desorption/Ionization Time-of-Flight Mass Spectrometry (MALDI-TOF MS) performed on a Microflex spectrometer (Bruker Daltonics, Bremen, Germany) using the Bruker MALDI Biotyper database (version 3.0).

Minimum inhibitory concentrations (MICs) for meropenem and other selected antimicrobial agents were determined using gradient diffusion (E-test; bioMérieux, Marcy-l’Étoile, France) in accordance with the manufacturer’s instructions and routine clinical laboratory protocols.

Given the absence of EUCAST or CLSI clinical breakpoints for carbapenems in *Campylobacter jejuni*, MIC values were not interpreted using standardized susceptibility categories. For fluoroquinolones and macrolides, EUCAST breakpoints were used. All isolates were tested in the same laboratory using consistent methodology to ensure comparability across episodes.

Whole-genome-sequencing (WGS), assembly, annotation and resistance gene prediction

Genomic DNA was extracted from overnight plate cultures using DNeasy Blood & Tissue Kits (Qiagen, Hilden, Germany). Illumina NovaSeq 6000 sequencing (Illumina Inc., CA, USA) was used to generate and assemble 150-bp paired-end reads. An average sequencing depth of 200× was achieved for the genomes. Genomes were assembled with SPAdes version 3.14.0 (Bankevich, Nurk et al. 2012).Genome annotation was carried out using Prokka(Seemann 2014). Resfinder (http://genepi.food.dtu.dk/resfinder) were used to screen acquired antimicrobial resistance (AMR) determinants. STs (sequence types) were determined with PubMLST *Campylobacter jejuni/coli* database (https://pubmlst.org/organisms/campylobacter-jejunicoli).

Snippy (v4.6.0) (https://github.com/tseemann/snippy) was used to call Single Nucleotide Polymorphisms (SNPs) and insertions/deletions (indels) between the reference Prokka-annotated GenBank (gbk) file of index isolate of each patient episode and subsequent isolates with raised meropenem MICs. Snippy default parameters, with a minimum depth coverage threshold of 10 reads for variant calling was employed.

Phylogenetic analysis

Performed usjing CSI Phylogeny 1.4 <https://cge.food.dtu.dk/services/CSIPhylogeny/>. A separate patient isolate *Campylobacter jejuni* with sequence type 9600-like was used as the reference genome. The output tree was visualized with the interactive tree of life (iTOL) (Letunic and Bork 2016). SNP matrix output from NASP was generated using snp-dists (https://github.com/tseemann/snp-dists).

Protein modeling and mutation identification

Whole-genome sequencing (WGS) was performed for all isolates. Non-synonymous mutations in key resistance-associated proteins, including penicillin-binding protein 3 (PBP3), major outer membrane porin (PorA), and class D β-lactamase OXA-193, were identified by comparative genomic analysis against each patient’s susceptible index isolate using Snippy (v4.6) and Prokka annotations.

Structural prediction and stability analysis

Protein models were generated using AlphaFold2 (Abramson, Adler et al. 2024) reference structures or homologous templates from the Protein Data Bank (PDB) when available. The structural and energetic effects of amino acid substitutions were predicted using DynaMut2 https://biosig.lab.uq.edu.au/dynamut2/(Rodrigues, Pires et al. 2021) and FoldX (https://foldxsuite.crg.eu/node/196). Predicted ΔΔG (kcal·mol⁻¹) values and qualitative stability changes were summarized for each mutation from both computational tools. Results were compared between isolates with raised meropenem MICs and their susceptible index isolates to assess whether predicted destabilizing substitutions may contribute to altered β-lactam susceptibility.


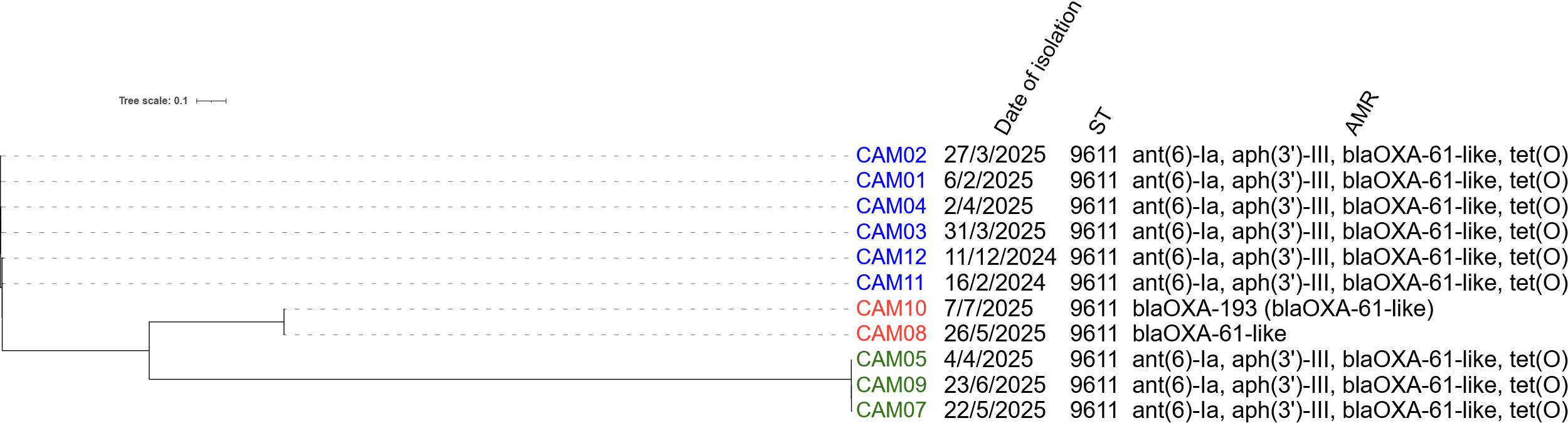


**Supplementary Figure 1**. SNP-based phylogenetic tree of longitudinal *Campylobacter jejuni* isolates from three patients. Phylogenetic analysis was performed using CSI Phylogeny v1.4 (https://cge.food.dtu.dk/services/CSIPhylogeny/), with a *C. jejuni* sequence type (ST) 9600-like isolate from a separate patient used as the reference genome. Isolates from Patient 1 (blue), Patient 2 (green), and Patient 3 (red) are shown with associated metadata including date of isolation (day/month/year), sequence type, and acquired antimicrobial resistance determinants. The scale bar (0.1) represents the number of substitutions per site.
